# Current Knowledge on the Role of Cardiolipin Remodeling in the Context of Lipid Oxidation and Barth Syndrome

**DOI:** 10.3389/fmolb.2022.915301

**Published:** 2022-05-27

**Authors:** Zhuqing Liang, Michael W. Schmidtke, Miriam L. Greenberg

**Affiliations:** Department of Biological Sciences, Wayne State University, Detroit, MI, United States

**Keywords:** cardiolipin remodeling, oxidation, apoptosis, barth syndrome, cardiolipin

## Abstract

Barth syndrome (BTHS, OMIM 302060) is a genetic disorder caused by variants of the *TAFAZZIN* gene (G 4.5, OMIM 300394). This debilitating disorder is characterized by cardio- and skeletal myopathy, exercise intolerance, and neutropenia. TAFAZZIN is a transacylase that catalyzes the second step in the cardiolipin (CL) remodeling pathway, preferentially converting saturated CL species into unsaturated CLs that are susceptible to oxidation. As a hallmark mitochondrial membrane lipid, CL has been shown to be essential in a myriad of pathways, including oxidative phosphorylation, the electron transport chain, intermediary metabolism, and intrinsic apoptosis. The pathological severity of BTHS varies substantially from one patient to another, even in individuals bearing the same *TAFAZZIN* variant. The physiological modifier(s) leading to this disparity, along with the exact molecular mechanism linking CL to the various pathologies, remain largely unknown. Elevated levels of reactive oxygen species (ROS) have been identified in numerous BTHS models, ranging from yeast to human cell lines, suggesting that cellular ROS accumulation may participate in the pathogenesis of BTHS. Although the exact mechanism of how oxidative stress leads to pathogenesis is unknown, it is likely that CL oxidation plays an important role. In this review, we outline what is known about CL oxidation and provide a new perspective linking the functional relevance of CL remodeling and oxidation to ROS mitigation in the context of BTHS.

## Introduction

As the hallmark lipid of the mitochondrial inner membrane, cardiolipin (CL) is essential for a myriad of cellular functions. CL undergoes a unique and highly regulated remodeling process catalyzed in part by the enzyme TAFAZZIN (Vreken et al., 2000). Variations in TAFAZZIN lead to the life-threatening disease Barth syndrome (BTHS), underscoring the importance of CL homeostasis and remodeling for cellular and organismal fitness (D'Adamo et al., 1997; Xu et al., 2003).

Newly synthesized CL contains predominantly saturated fatty acid chains. The primary outcome of CL remodeling is the incorporation of polyunsaturated fatty acids (PUFAs) into CL molecules. Unlike saturated and monounsaturated fatty acids, PUFAs are susceptible to oxidation in the presence of elevated reactive oxygen species (ROS), and oxidation of PUFA-CL has been shown to underlie cellular sensitivity to apoptosis (Belikova et al., 2006). Recent studies have demonstrated that oxidized CL (CL_ox_) forms a complex with the intermembrane space (IMS) protein cytochrome *c* (cyt *c*), and that this complex catalyzes the oxidation of additional PUFA-CL molecules (Kagan et al., 2005).

Here we describe a new perspective for understanding the evolutionarily conserved role of CL remodeling in the context of CL oxidation and BTHS. This model represents a paradigm shift for BTHS research by identifying a novel link between TAFAZZIN function and BTHS pathophysiology. Future studies should be aimed at improving our understanding of this model and evaluating the potential for treating BTHS by inhibiting this oxidation pathway.

## What is CL?

CL is a uniquely dimeric phospholipid found almost exclusively in mitochondria. It is comprised of a glycerol backbone bridging two canonical phosphatidylglycerol molecules. The variable structure of the four associated fatty acid chains, along with the negative charge carried by the two phosphate head groups, are important for the many protein-lipid interactions attributed to CL (Planas-Iglesias et al., 2015). Following its initial synthesis, CL undergoes a remodeling process in which multiple cycles of deacylation and reacylation generate predominantly mono- and polyunsaturated fatty acid-containing CL (PUFA-CL). In mammals, this generally involves replacing oleic acid (18:1) acyl chains with linoleic acid (18:2) to form tetralinoleoyl-CL (Xu et al., 2006; Oemer et al., 2020). The importance of the CL remodeling pathway is underscored by the severe disease BTHS, in which patients bear variations in the CL-specific transacylase TAFAZZIN (Barth et al., 1999). At the cellular level, TAFAZZIN deficiency causes a decrease in total CL levels, an aberrant CL profile characterized by a decrease in unsaturated CL, and accumulation of monolyso-CL (mCL), biochemical hallmarks that have been observed in all BTHS models tested to date (Vreken et al., 2000; Valianpour et al., 2002; Schlame et al., 2003; Gu et al., 2004; Houtkooper et al., 2006; Acehan et al., 2011). Although the pathological consequences of TAFAZZIN deficiency have been well-characterized in BTHS patients, the exact mechanism(s) linking defective CL remodeling to these phenotypes remain elusive.

TAFAZZIN is the primary enzyme responsible for reacylation of CL in healthy cells, but two additional acyltransferases have been described in mammals. Similar to TAFAZZIN, both of these enymzes, acyl-CoA:lysocardiolipin acyltransferase 1 (ALCAT1) and monolysocardiolipin acyltransferase 1 (MLCL AT-1), preferentially transfer oleic and linoleic acid acyl chains to CL, generating predominantly unsaturated CL species (Ma et al., 1999; Taylor and Hatch, 2003; Cao et al., 2004; Cao et al., 2009). Interestingly, overexpression of ALCAT1 has been shown to promote ROS production whereas overexpression of MLCL AT-1 attenuates ROS production in BTHS lymphoblasts (Li et al., 2010; Mejia et al., 2018). Given their secondary role to TAFAZZIN, the physiological relevance of each of these enzymes remains unclear, and adding to this complexity, it has been shown that MLCL AT-1 expression is upregulated by TAFAZZIN knockdown in healthy cells but not in BTHS lymphoblasts (Mejia et al., 2018). Thus, further studies are needed to determine the relative contributions of these enzymes to PUFA-CL production in both healthy cells and BTHS tissues.

## PUFA-CL Likely Confers a Fitness Advantage in Mammalian Cells

In mammalian cells, it is likely that remodeled CL confers a fitness advantage. Intact CL remodeling has been shown to be important for various aspects of cellular and mitochondrial homeostasis, including regulation of mitochondrial dynamics, induction of mitophagy and apoptosis, protein turnover, and calcium uptake (Chu et al., 2013; Hsu et al., 2015; Ban et al., 2017; de Taffin de Tilques et al., 2018; Kameoka et al., 2018; Petit et al., 2020; Bertero et al., 2021). In human heart and skeletal muscle mitochondria, 80% of all endogenous CL is tetralinoleoyl-CL, and decreased tetralinoleoyl-CL has been associated with aging and sarcopenia (Sparagna et al., 2007; Chu et al., 2013; Oemer et al., 2018; Semba et al., 2019; Zhang et al., 2022). Thus, defective CL remodeling in BTHS results in a broad range of cellular deficiencies that may contribute to the pathophysiology.

Although the above findings demonstrate the general importance of CL remodeling, the exact nature of how PUFA-CL benefits cells is not clear. One molecular hypothesis is that protein-lipid packing in the inner mitochondrial membrane (IMM) imposes mechanical stress that favors the accumulation of PUFA-CL, which can only be generated through remodeling. Due to its intrinsic negative curvature, membranes enriched with PUFA-CL can accommodate a higher density of electron transport chain (ETC) complexes and thereby act as more efficient sites for oxidative phosphorylation (OXPHOS) (Musatov, 2006; Schlame and Xu, 2020). However, due to the presence of multiple carbon-carbon double bounds, PUFA-CL is vulnerable to being oxidized by ROS produced by the ETC, and as detailed below, this susceptibility has important consequences for maintaining cellular homeostasis.

## Oxidation of PUFA-CL is Detrimental to Cell Homeostasis

In healthy cells, energy production *via* OXPHOS relies on the electron shuttling activity of cyt *c*, an IMS protein whose localization is maintained, in part, by binding with CL. The interaction between CL and cyt *c* is a critical determinant of cell homeostasis. Cyt *c* contains two CL binding sites; loose binding of CL to the “A-site” is mediated by reversible electrostatic interactions between positively charged lysine residues 54/55, 72/73, 86/87, and the negatively charged head groups of CL, whereas tighter binding to the “C-site” involves hydrophobic residues in the fatty acyl chains of CL (Rytomaa et al., 1992; Rytomaa and Kinnunen, 1994; Kagan et al., 2005; Gonzalvez and Gottlieb, 2007; Elmer-Dixon and Bowler, 2018). In the loosely bound conformation mediated by site A, cyt *c* conducts its canonical function of transferring electrons from ETC complex III to complex IV. Conversely, when the C-site is bound, cyt *c* becomes partially unfolded. This exposes a ROS binding site on the associated heme, which effectively converts the CL-cyt *c* complex into a potent lipid peroxidase that preferentially oxidizes PUFA-CL to form CL_ox_ (Kagan et al., 2005; Belikova et al., 2006; Kagan et al., 2014). Due to its localization in the IMM, CL is highly prone to oxidation, as ETC complex activity is a major source of ROS production even in healthy cells (Raha et al., 2000; Koopman et al., 2010; Holzerova and Prokisch, 2015).

Oxidation of CL facilitates apoptosis by disrupting binding and localization of cyt *c*. Binding between cyt *c* and CL has been shown to depend on the oxidation status of CL. Specifically, cyt *c* has a lower affinity for CL_ox_ than non-oxidized CL, and thus CL oxidation results in increased release of cyt *c* from the IMS, a key event that activates the mitochondria-mediated apoptosis pathway, and increased sensitivity to apoptotic stimuli (Nakagawa, 2004; Jiang et al., 2008; Tyurina et al., 2012). The release of cyt *c* from the IMS takes place in two steps: cyt *c* detachment from CL_ox_, followed by the permeabilization of the outer mitochondria membrane (OMM). The reduced affinity of cyt *c* for CL_ox_ likely contributes to detachment of the former from the IMM, though the exact biochemistry of this process remains obscure. Subsequent translocation of cyt *c* into the cytosol requires permeabilization of the OMM. The mechanism(s) responsible for OMM permeabilization are the subject of much debate, but two potential explanations have received considerable attention (Robertson et al., 2003; Gogvadze et al., 2006). In the first scenario, calcium overload in the matrix promotes formation of a permeability transition pore in the IMM, which results in swelling and eventual rupture of the OMM (Gogvadze et al., 2006). In the second scenario, the Bcl-2 family protein Bax is recruited to the OMM by tBid where it then homo-oligomerizes to form a pore through which cyt *c* can diffuse. Regardless of the exact mechanism, cyt *c* released from the mitochondria stimulates apoptosis by triggering oligomerization of apoptosis protease activating factor-1 and subsequent activation of pro-caspase-9 (Yu et al., 2005). Interestingly, reduced levels of PUFA-CL in BTHS cells have been linked to a defect in induction of apoptosis, owing to diminished binding affinity between unremodeled CL and caspase-8 (Gonzalvez et al., 2008; Gonzalvez et al., 2013). This suggests that CL remodeling plays an important role in balancing sensitivity to apoptosis, as a decrease in PUFA-CL leads to deficient apoptotic induction while the production of CL_ox_ from PUFA-CL results in hypersensitivity to apoptosis.

The production of CL_ox_ has other important consequences. CL in the IMM acts to bind and stabilize ETC complexes, but when CL becomes oxidized, this interaction is diminished, leading to a concomitant reduction in ETC function (Musatov, 2006). Recent work has also shown that the yeast CL phospholipase Cld1 has a greater affinity for CL_ox_ than non-oxidized CL *in vitro* (Lou et al., 2018b), suggesting that repeated cycles of CL remodeling in the presence of CL_ox_ preferentially generate oxidized (vs non-oxidized) free fatty acids (FA_ox_). FA_ox_, and highly reactive aldehydes readily derived from them (Esterbauer et al., 1991; Gueraud et al., 2010), have a variety of secondary messenger signaling roles relating to apoptotic induction (Iuchi et al., 2019), inflammation (Ramakrishnan et al., 2014; Dennis and Norris, 2015), metabolism (Hauck and Bernlohr, 2016; Wenzel et al., 2017), vascular regulation (Sudhahar et al., 2010), and calcium homeostasis (Saraswathi et al., 2004) among other functions (Buland et al., 2016). In light of these primarily negative consequences, selection pressure should favor adaptive mechanisms for preventing or mitigating the production of CL_ox_ in cells.

## Discussion and Perspective

The pathologies associated with BTHS illustrate the global importance of CL remodeling. However, the capacity for oxidation in the presence of ROS represents a potential drawback to the production of PUFA-CL through the CL remodeling pathway, and thus a complete understanding of why remodeling has been maintained through evolution remains elusive. This has been highlighted by studies indicating that remodeled and unremodeled CL are functionally equivalent in yeast, and that the pathology of BTHS stems from an elevated mCL:CL ratio rather than a lack of PUFA-CL *per se* (Baile et al., 2014; Ye et al., 2014).

The predominance of PUFA-CL (most notably tetralinoleoyl-CL) suggests that it confers a fitness advantage in mammalian cells, and CL remodeling is often thought of solely as a mechanism for converting saturated CL into PUFA-CL. However, if CL remodeling only functions to generate PUFA-CL, why have cells not evolved to synthesize PUFA-CL directly, rather than depend on the additional multi-step process of converting nascent, primarily saturated CL to PUFA-CL through remodeling? Indeed, it has been shown that exogenously obtained PUFAs can be readily incorporated into newly synthesized CL, indicating that remodeling for the sole purpose of producing PUFA-CL would be redundant when PUFAs can be dietarily acquired (Tyurina et al., 2017). This suggests that the CL remodeling pathway may serve an additional function, and a hint regarding this comes from the study by Lou et al., showing that the yeast CL phospholipase Cld1, which mediates the first step in the CL remodeling pathway, exhibits a clear preference for removing oxidized acyl chains from CL_ox_ (Lou et al., 2018b). Furthermore, this study demonstrated that expression of the *CLD1* gene is upregulated in response to H_2_O_2_ treatment, suggesting that Cld1 function is regulated as a homeostatic response to elevated ROS (Lou et al., 2018b). Taken together, these findings support the novel hypothesis that CL remodeling may serve as a conserved, compensatory mechanism for removing oxidized fatty acid chains from CL_ox_ in order to mitigate oxidative damage and regenerate non-oxidized PUFA-CL in the second TAFAZZIN-catalyzed step of the remodeling process.

In view of this model, one point should be noted. The notion that CL remodeling has evolved as a mechanism to remove CL_ox_ and regenerate non-oxidized CL presupposes that the phenotype of having elevated CL_ox_ is more deleterious than generating FA_ox_ and associated reactive aldehyde species. This point is supported by the fact that cells have robust mechanisms for neutralizing FA_ox_/aldehydes, including adduction with glutathione, reduction by aldo-keto reductases or alcohol dehydrogenases, and oxidation by aldehyde dehydrogenases (Gueraud et al., 2010; Pizzimenti et al., 2013), whereas the diminished function of CL_ox_ causes widespread problems related to cell viability and bioenergetics that cannot be mitigated efficiently.

In the context of BTHS, this hypothesis may partially explain the deleterious consequences of TAFAZZIN deficiency. Although PUFA-CL is primarily produced through the CL remodeling pathway in healthy cells, it is important to note that PUFAs can be incorporated into nascent CL when PUFA-containing precursor lipids are available (e.g., from nutritionally-derived sources) (Tyurina et al., 2017; Lou et al., 2018b). This means that even in TAFAZZIN-deficient cells, oxidizable PUFA-CL will be present, albeit in relatively low abundance compared to wild type cells. Elevated ROS is a characteristic of BTHS models, and this likely facilitates oxidation of the existing PUFA-CL by CL-cyt *c* peroxidase complexes (Chen et al., 2008; Lou et al., 2018a; Liu et al., 2021). The key difference between wild type and TAFAZZIN-deficient cells would become apparent in the homeostatic response to elevated CL_ox_. While wild type cells would be capable of upregulating their remodeling pathway by increasing iPLA2γ phospholipase expression and relying on TAFAZZIN to reacylate mCL with predominantly non-oxidized PUFAs, TAFAZZIN-deficient cells might still upregulate iPLA2γ phospholipase expression but would be incapable of regenerating PUFA-CL.

In summary, this novel paradigm posits that CL remodeling has been evolutionarily conserved as a means for mitigating the deleterious effects of CL_ox_ production. As an extension of this framework, a major pathophysiological outcome of TAFAZZIN deficiency in BTHS is the inability of cells to recycle CL_ox_ and regenerate non-oxidized PUFA-CL. Future studies should be aimed at testing this model, as it may suggest a new avenue for treating BTHS.

## Data Availability

The original contributions presented in the study are included in the article/Supplementary Material, further inquiries can be directed to the corresponding author.
